# Brain function effects of autonomous sensory meridian response (ASMR) video viewing

**DOI:** 10.3389/fnins.2023.1025745

**Published:** 2023-01-26

**Authors:** Noriko Sakurai, Kazuaki Nagasaka, Shingo Takahashi, Satoshi Kasai, Hideaki Onishi, Naoki Kodama

**Affiliations:** ^1^Department of Radiological Technology, Niigata University of Health and Welfare, Niigata, Japan; ^2^Department of Physical Therapy, Niigata University of Health and Welfare, Niigata, Japan; ^3^Department of Healthcare Informatics, Takasaki University of Health and Welfare, Gunma, Japan

**Keywords:** ASMR, audiovisual stimulation, auditory stimulation, nucleus accumbens, fMRI

## Abstract

**Background:**

Autonomous sensory meridian response (ASMR) is the sensation of tingling from audiovisual stimuli that leads to positive emotions. ASMR is used among young people to relax, induce sleep, reduce stress, and alleviate anxiety. However, even without experiencing tingling, ASMR is used by many young people to seek relaxation. Auditory stimulation in ASMR is thought to play the most important role among its triggers, and previous studies have used a mixture of auditory and visual stimulation and auditory stimulation. This is the first study to approach the differences between the effects of direct audiovisual and auditory stimulation from the perspective of brain function using functional magnetic resonance imaging (fMRI) and to clarify the effects of ASMR, which attracts many young people.

**Methods:**

The subjects were 30 healthy subjects over 19 years old or older who had not experienced tingling. Brain function was imaged by fMRI while watching ASMR videos or listening to the sound files only. We administered a questionnaire based on a Likert scale to determine if the participants felt a “relaxed mood” and “tingling mood” during the task.

**Results:**

Significant activation was found in the visual cortex for audiovisual stimulation and in the visual and auditory cortex for auditory stimulation. In addition, activation of characteristic sites was observed. The specific sites of activation for audiovisual stimulation were the middle frontal gyrus and the left nucleus accumbens, while the specific sites of activation for auditory stimulation were the bilateral insular cortices. The questionnaire showed no significant differences in either “relaxed mood” or “tingling mood” in response to auditory and visual stimulation or auditory stimulation alone.

**Conclusion:**

The results of this study showed that there was a clear difference between auditory and audiovisual stimulation in terms of the areas of activation in the brain, but the questionnaire did not reveal any difference in the subjects’ mood. Audiovisual stimulation showed activation of the middle frontal gyrus and the nucleus accumbens, whereas auditory stimulation showed activation of the insular cortex. This difference in brain activation sites suggests a difference in mental health effects between auditory and audiovisual stimulation. However, future research on comparisons between those who experience tingling and those who do not, as well as investigations of physiological indices, and examination of the relationship with activated areas in the brain may show that ASMR is useful for mental health.

## 1. Introduction

Autonomous sensory meridian response (ASMR) is a tingling somatosensory phenomenon that begins in the scalp and extends to the back of the neck, arms, and legs (Barratt and Davis, 2015). Many videos that elicit ASMR, which are becoming increasingly popular among young people, have been posted on YouTube and other social media sites so that people can experience the sensation (Barratt and Davis, 2015; Barratt et al., 2017). This somatic somatosensory is reported to be more likely to be experienced by open-minded individuals based on personality trait analysis (Fredborg et al., 2017). It is believed that to experience tingling, one would wear headphones and choose a quiet, calm place, which is a very limited time and place. However, feeling relaxed and positive even when no tingling occurred, suggesting possible relief of chronic pain and coping with anxiety (Barratt and Davis, 2015; Morales et al., 2021). ASMR is used by many young people to seek relaxation, even if they do not experience tingling. The videos are created by emulating a wide variety of real-life experiences, such as tapping sounds, crisp sounds, nature sounds, soft whispers, and role-playing actions. Triggers of ASMR have been grouped into five categories (Watching, Touching, Repetitive Sounds, Simulations, and Mouth Sounds). However, some triggers do not fall into any of these categories, as ASMR is a highly personal and wide-ranging event (Fredborg et al., 2017).

The effects of ASMR have both a relaxing and calming aspect and an exciting and stimulating aspect. Lochte et al. (2018) evaluated people viewing ASMR videos with functional magnetic resonance imaging (fMRI) and reported brain activity during tingling. The results indicate that it is involved in the reward system, social behavior, and empathy, suggesting that the ASMR videos activate brain regions previously observed during experiences like social bonding and musical frisson. In terms of physiological responses, some individuals experienced increased skin conductance responses (a measure of autonomic nervous system arousal) and decreased heart rate (Poerio et al., 2018) when viewing ASMR videos. Valtakari et al. (2019) found that tingling causes pupil dilation, a physiological reaction toward ASMR stimulation that is similar to the increase in the skin conductance response. In electroencephalography (EEG), an increase in alpha waves in the frontal lobes of experienced ASMR subjects and a decrease in non-experienced subjects suggest the possibility of opposite effects among users (Engelbregt et al., 2022).

As for its relaxing and calming aspect, there are many reports that ASMR affects stress reduction, sleep, and relaxation. ASMR has been proposed as a way to improve sleep quality (Lee et al., 2019; Vardhan et al., 2020). This sleep induction is limited to auditory stimulation and uses natural sounds as triggers that are not likely to induce tingling. EEG measurements of the transition to sleep with combined auditory stimulation of binaural beats suggest that this combined stimulation may help in the transition to sleep (Lee et al., 2019). Cash et al. (2018) reported a placebo effect of ASMR, suggesting that a possible stress reduction effect can be induced by a somatosensory response with a 5-min audio file. ASMR is an efficient way to relax people’s minds and allows for easy home-based stress and pain management. Paszkiel et al. (2020) investigated the effects of four types of sound stimuli on stress levels based on four parameters (EEG, blood pressure, pulse, and questionnaire) and reported that both relaxing music and ASMR induce relaxation at a faster rate and have an effect on stress level reduction. We also used fMRI to elucidate the relaxing effects of ASMR on brain function. To compare with classical music, we focused our experiment on the ASMR sound file alone. In both cases, activation of the thalamus and other areas involved in sleep was observed. In particular, activation of the medial prefrontal cortex was observed in ASMR, suggesting that it is involved in the induction of relaxation (Sakurai et al., 2021).

Thus, Previous studies have reported on the effects of ASMR on relaxation, but they have reported only video or only sound evaluations. As a motivation for using ASMR, Barratt and Davis (2015) reported that ASMR helped 98% of participants to relax, 82% to sleep, 70% to cope with stress, and 80% to have a positive effect on mood. In a survey study, ASMR videos showed potential to improve mood and alleviate symptoms of insomnia and depression (Smejka and Wiggs, 2022). Poerio et al. (2018) also associated ASMR experiences with increased positive mood and relaxation. These reports of research on motivations for use are also about ASMR as a whole and do not clearly distinguish between audiovisual and auditory stimulation. Auditory stimulation of ASMR plays the most important role among the triggers, and auditory stimulation is reported to be more triggering than visual stimulation (Barratt et al., 2017). In our previous study, we compared the relaxation effects of ASMR sound alone with classical music as a control (Sakurai et al., 2021). Based on this further, the present study examined the brain activity of ASMR with images, in addition to the sound alone. However, there are no reports directly examining the comparative However, there are no reports directly examining the comparative effects of audiovisual and auditory stimulation. Furthermore, since ASMR is used by many young people to seek relaxation even if they have not experienced tingling, we decided to target those who have never experienced tingling. Therefore, the effectiveness of ASMR, which many young people are seeking, can be verified. To the best of the authors’ knowledge, this is the first study to clarify the effect of ASMR in attracting many young people based on the difference in brain function mechanisms between audiovisual and auditory stimuli of ASMR using fMRI.

## 2. Materials and methods

### 2.1. Participants

Thirty healthy subjects (15 males and 15 females, mean age 20.8 ± 0.5 years) aged 19 years and older, who had viewed ASMR videos but had never experienced tingling. They also had no history of psychiatric disorders, and were not taking any medications. In order to avoid the effects of habituation, the subjects were not allowed to watch ASMR at all for 1 week prior to the start of the experiment. Tingling was defined in this study as a creeping somatic sensation that originates in the scalp and extends from the back of the neck to the back or limbs. This study was approved by the Research Ethics Committee of Niigata University of Health and Welfare (approval No. 18218-190722). We provided informed consent to all subjects and obtained written consent. We also interviewed the subjects to ensure the safety of MRI imaging.

### 2.2. Stimuli task

The subjects were asked to select their preferred ASMR as the stimulus task. Five ASMRs of repetitive, crisp, and refreshing sounds (are scratching, eating cucumber, typing, pouring soda water, and rain sound) were selected from among the 10 used in our previous study. We excluded those that emphasized activity in the visual cortex, such as touching, because ASMR also can also be induced by a sound file alone.

Participants listened to all five types at least 1 week before the experiment and selected the one ASMR type that was most to their preference. We prepared a video of the selected ASMR and a sound file alone from that video. Editing was done using the video editing software Power Director 18 (Cyber Link Corporation) to create compressed audio data in MP4, and the volume was set at 98 dB for all participants. While listening to the sound file alone, the participant viewed a fixed white cross on a black screen.

The resting task prior to the stimulus task was listening to white noise. White noise was used because it is a random signal with equal power at any frequency within a given bandwidth that does not stimulate emotional involvement (Cardona et al., 2020). The same fixed crosses were displayed on the screen during the resting task.

### 2.3. Block design

**FIGURE 1:**
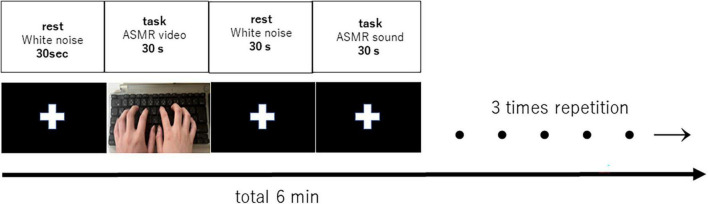
Block design of 6 min with 30 s rest and 30 s task (auditory and visual stimulation, auditory stimulation), repeated three times. Each subject listened or viewed the tasks in random order.

A block design was used to alternate between the resting task and the stimulus task for 30 s each, for a total of 6 min. White noise and fixed crosses were included during the resting task to separate the stimulation time from the baseline blood oxygenation signal from the fMRI. The block design is shown in Figure 1.

### 2.4. Apparatus

Imaging was performed using a 3-tesla MRI system (Vantage Galan, Canon Medical Systems, Tochigi, Japan) with a 16-channel head coil. The participant laid down in the MRI machine and watched the block design. The images were displayed on the MR-theater (Canon, Inc.) and could be viewed while lying down. The sound source was an MRI headphone system (Star Products, Inc., iMage), which has high sound insulation and blocks out scanning sounds.

### 2.5. MRI Acquisition

High-resolution MRI images were captured separately to obtain detailed anatomical information before fMRI imaging. The structural image for this purpose was a high-resolution T1-weighted magnetization-prepared rapid-gradient-echo (MP-RAGE) sequence. The settings were: repetition time (TR) = 5.8 ms, echo time (TE) = 2.7 ms, inversion time (TI) = 900 ms, flip angle (FA) = 9°, number of matrix (matrix) = 256 × 256, field of view (FOV) = 23 × 23 cm, slice thickness = 1.2 mm. Echo-planar imaging (EPI) sequences were used to capture fMRI images. The imaging conditions for the fMRI images were as follows: TR = 2,000 ms, TE = 25 ms, FA = 85°, matrix = 64 × 64, FOV = 24 × 24 cm, and slice thickness = 3 mm so that the entire brain could be covered.

### 2.6. fMRI data analyses

The fMRI data were preprocessed and analyzed using Statistical Parametric Mapping 12 (Wellcome Trust Center for Neuroimaging) running on Matlab (Mathworks Inc.). After correcting for time differences with slice timing correction, displacement due to motion was corrected with realignment. In addition, coregisters were used to compare structural and fMRI images. The coregister corrected for displacement between structural and functional images and normalized the data by aligning each participant’s brain with the Montreal Neurological Institute’s standard brain coordinate system template. Normalized images were smoothed using an 8 mm Gaussian kernel. After preprocessing, changes in brain activity associated with audiovisual and auditory stimulation of ASMR were identified by block design using the general liner model (GLM). A head movement parameter was added to remove the effect of movement. Contrast images were produced at the first level (single subject) with the following contrasts: (1) ASMR audiovisual stimulation = 1, rest = 0, (2) ASMR auditory stimulation = 1, rest = 0, (3) ASMR audiovisual stimulation = 1, ASMR auditory stimulation = −1, and (4) ASMR audiovisual stimulation = −1, ASMR auditory stimulation = 1, respectively. The contrasts in (3) were used to identify brain regions that showed significantly increased activity with audiovisual stimulation compared to the auditory stimulation condition. The contrasts in (4) were used to identify brain regions that showed significantly increased activity with auditory stimulation compared to the audiovisual stimulation condition. In the next group analysis (second level), a one-sample *t*-test was performed using the above four contrasts. The initial threshold for voxel level was set at uncorrected *p* < 0.001. Clusters were considered significant if they were below *p* = 0.05 corrected for family wise errors. Audiovisual and auditory stimulation of ASMR were analyzed separately; audiovisual stimulation minus auditory stimulation and auditory stimulation minus audiovisual stimulation was subtracted from audiovisual stimulation.

### 2.7. Questionnaire

We administered a mood questionnaire to all subjects after fMRI imaging. We investigated two different mood intensities for each ASMR for audiovisual and auditory stimulation. The two moods were “relaxed mood” and “tingling mood,” using a Likert-type scale from 1 to 5: (1) totally disagree, (2) disagree, (3) neither agree nor disagree, (4) agree, (5) agree very much. The subjects were told that “relaxed mood” refers to a state of calm and peacefulness. Statistical analysis was conducted using SPSS (IBM SPSS Statistics Base) 26.0 with the Mann–Whitney U test. The significance level was set at 5%.

## 3. Results

The questionnaire results showed no significant difference between audiovisual and auditory stimulation of ASMR in terms of “relaxed mood” and “tingling mood.” The results are shown in Table 1.

**TABLE 1:** Questionnaire results for the two moods (“relaxed mood” and “tingling mood”).

Likert scale point		1	2	3	4	5	
Relaxed mood	Audiovisual stimulations	0 (0.00%)	1 (3.33%)	4 (13.33%)	18 (60.00%)	7 (23.33%)	*p* = 1.000
	Auditory stimulations	0 (0.00%)	1 (3.33%)	4 (13.33%)	18 (60.00%)	7 (23.33%)	
Tingling mood	Audiovisual stimulations	7 (23.33%)	4 (13.33%)	3 (10.00%)	14 (46.67%)	2 (6.67%)	*p* = 0.895
	Auditory stimulations	6 (20.00%)	4 (13.33%)	5 (16.67%)	12 (40.00%)	3 (10.00%)	

**FIGURE 2:**
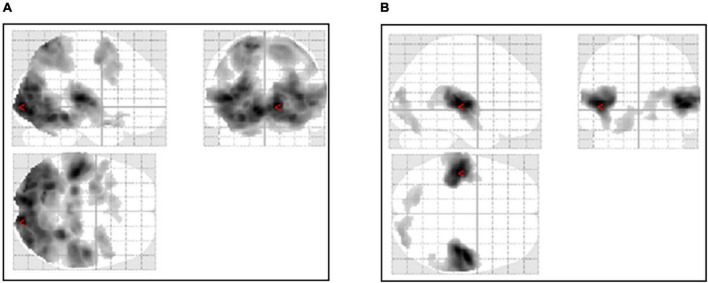
Activation of the whole brain during the stimulation task. **(A)** Activation during the ASMR audiovisual stimulation task. **(B)** Activation during the ASMR auditory stimulation task.

Audiovisual stimulation of ASMR showed activation of the visual cortex, the left and right middle frontal gyrus, and the nucleus accumbens. Auditory stimulation showed activation of the auditory and visual cortices and the left and right insular cortices. Table 2 shows the coordinates of the areas that were significantly activated during the stimulation task. Figure 2 shows a glass-brain image of the activation of the whole brain during the stimulation task.

**TABLE 2:** Significantly activated areas and *T*-values of stimulation task performance.

Hemisphere		Locations	Cluster *p*-value (FWE)	Cluster size (voxels)	*T*-value	Z-score	X {mm}	Y {mm}	Z {mm}
Audiovisual stimulations	Right	Calcarine cortex	<0.001	26,869	13.49	7.53	12	−92	0
	Left	Middle frontal gyrus	<0.001	1,024	7.32	5.47	−26	−4	48
	Right	Middle frontal gyrus	<0.001	960	6.62	5.13	32	4	66
	Left	Accumbens area	0.009	419	6.05	4.83	−14	4	−20
Auditory stimulations	Left	Insula	<0.001	2,536	11.82	7.09	−46	−22	4
	Right	Insula	<0.001	2,648	11.68	7.05	50	−14	4
	Right	Occipital pole	0.001	593	5.43	4.48	24	−90	16
	Left	Lingual gyrus	0.005	459	4.81	4.09	−14	−74	−18

**FIGURE 3:**
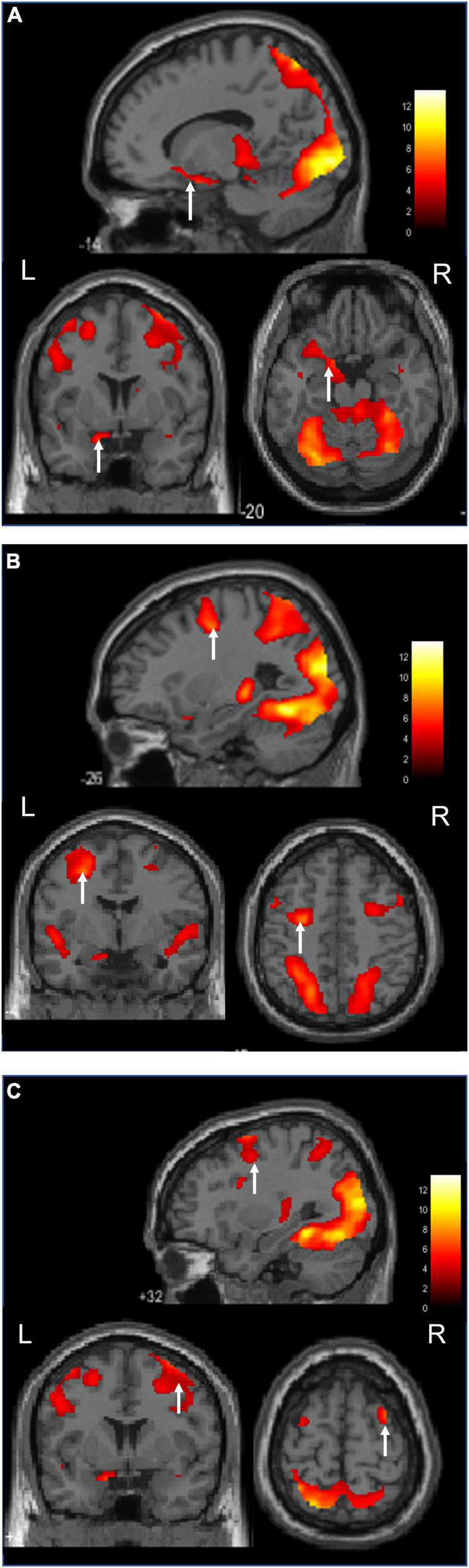
Activation during the ASMR audiovisual stimulation task (Sagittal, coronal, and axial images.). **(A)** Activation of the left nucleus accumbens is confirmed. **(B)** Activation of the left middle frontal gyrus is confirmed. **(C)** Activation of the right middle frontal gyrus is confirmed.

Figure 3 shows an image of the activation of the nucleus accumbens, which is a characteristic feature of the ASMR audiovisual stimulation task.

**FIGURE 4:**
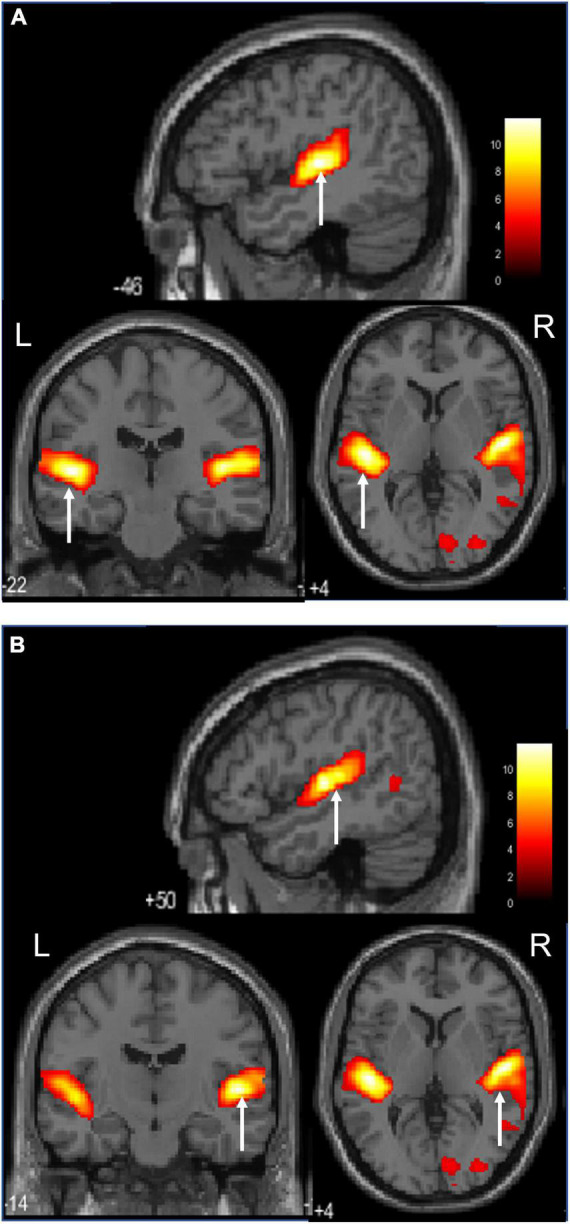
Activation during the ASMR audiovisual stimulation task (Sagittal, coronal, and axial images.).**(A)** Activation of the left insula cortices is confirmed. **(B)** Activation of the right insula cortices is confirmed.

Figure 4 shows images of the activation of the left and right insular cortices, which characteristic the ASMR auditory stimulation task.

The regions with significantly increased activity during audiovisual stimulation compared to auditory stimulation were the left lingual gyrus, left postcentral gyrus, left and right middle frontal gyrus, and left lateral nucleus accumbens. The regions of significantly increased activity during auditory stimulation compared to the audiovisual stimulation condition were the left middle occipital gyrus, right precuneus, right angular gyrus, and left lateral ventricle. Their *T*-values and coordinates are shown in Table 3.

**TABLE 3:** *T*-values and coordinates of the sites of significantly increased activity by audiovisual stimulation and auditory stimulation.

	Hemisphere	Locations	Cluster *p*-value (FWE)	Cluster size (voxels)	*T*-value	Z-score	X {mm}	Y {mm}	Z {mm}
Audiovisual stimulations > Auditory stimulations	Left	Lingual gyrus	<0.001	21,737	13.67	7.57	−10	−82	−10
	Left	postcentral gyrus	0.007	419	8.62	6.02	−54	−22	38
	Left	Middle frontal gyrus	<0.001	756	7.06	5.34	−24	−6	48
	Right	Middle frontal gyrus	0.015	351	5.57	4.55	28	0	68
	Left	Accumbens area	0.007	426	4.99	4.2	−14	6	−20
	Left	Middle frontal gyrus	0.007	420	4.65	3.99	−52	8	38
	Right	Middle frontal gyrus	0.067	226	4.62	3.97	36	10	26
Audiovisual stimulations < Auditory stimulations	Left	Middle occipital gyrus	0.001	675	9.10	6.21	−46	−68	32
	Right	Precuneus	<0.001	2,059	8.61	6.02	22	−44	22
	Right	Angular gyrus	<0.001	767	7.77	5.67	46	−64	42
	Left	Lateral ventricle	0.021	325	5.24	4.36	−22	−28	30

## 4. Discussion

This is the first fMRI-based brain function study focusing on the effects of ASMR video viewing compared with listening to sound file alone. The participants showed no difference in mood when they watched the ASMR video or listened to the sound file alone, but they revealed a difference in the brain function.

The audiovisual ASMR task stimulated the visual cortex, whereas the auditory task stimulated the auditory and visual cortices. As for the difference between visual and auditory stimulation, according to Norretranders, human sensory organs receive over 11.1 (Mbit/s) of external information, of which 10 (Mbit/s) is visual and 0.1 (Mbit/s) is auditory. The amount of information input to the brain per second for visual stimulation is said to be 100 times greater than that for auditory stimulation (Norretranders, 1998). It is thought that the greater amount of information from the visual stimuli during audiovisual stimulation focuses attention on movement. In the auditory task, the visual cortex was also activated because the participants were watching the presentation of the fixed cross, but it is thought that their attention was focused more on the sound. However, since the fixed crosses were identical during the resting time and the audio, we believe that they may not have been separated from the resting time. In the future, the experimental design for auditory stimulation needs to be improved and additional experiments should be conducted in the absence of a fixed cross during auditory stimulation. In addition to the visual and auditory cortices, activation was also observed in more characteristic areas. Audiovisual stimulation showed activation in the middle frontal gyrus and left temporal nucleus, which is not seen in other areas, and auditory stimulation showed activation in the bilateral insular cortex. Lochte et al. (2018) reported brain function during tingling while viewing ASMR videos; subjects experiencing ASMR showed significant activation in the nucleus accumbens, dorsal anterior cingulate cortex, insular cortex, and inferior frontal gyrus, and these are regions associated with both reward and emotional arousal. Although the block design differed from the present study, the results were similar, if not tingling.

Audiovisual stimulation of the middle frontal gyrus and nucleus accumbens plays an important role in the mesolimbic dopamine pathway, which operates as a reward mechanism. Dopaminergic input from the ventral tegmental area connects to the limbic system, including the nucleus accumbens, and to other structures, such as the prefrontal cortex and amygdala. The nucleus accumbens is an important brain region for reward, gratification, and emotion and is largely responsible for the release of dopamine (Pereira et al., 2011). It is also involved in improving pain, depression, and anxiety symptoms (Du et al., 2017; Harris and Peng, 2020).

The middle frontal gyrus involvement was bilateral, while the nucleus accumbens was left-sided. Du et al. (2017) reported that the strength of connectivity between the left dorsolateral prefrontal cortex and the left nucleus accumbens was negatively correlated with improvement in depressive and anxiety symptoms. The activation of the left middle frontal gyrus and left nucleus accumbens in the present results suggests a sufficient involvement in depression and anxiety. From the results of differential ASMR audiovisual and auditory stimulation, the left and right middle frontal gyrus and left temporal nucleus were identified as the regions significantly activated by audiovisual stimulation. The involvement of the mesolimbic dopamine circuitry seems certain. Smejka and Wiggs (2022) also reported that ASMR movies might improve mood and alleviate symptoms of insomnia and depression. These reports are also considered encouraging from the viewpoint of brain function. In addition, subtraction of audiovisual and auditory stimuli in ASMR revealed that audiovisual stimulation significantly activated the left and right middle frontal gyrus and left lateral nucleus. The involvement of mesolimbic dopamine pathways seems certain.

The left and right insular cortices activated by auditory stimulation are adjacent to the primary auditory cortex, which is essential for human cognitive behavior and is involved in sensorimotor activity, pain, empathy, and high levels of attention and decision making (Uddin et al., 2017). In particular, the posterior insula is closely associated with the auditory cortex and is involved in the network for receiving and processing auditory information. The anterior insula is also involved in processing autonomic information and in perceiving the emotional states of others and feeling those emotions yourself (Zhang et al., 2019). Thus, the insular cortex is involved in integrating internal and external information and is present to form a global perception of how the self feels (Craig, 2009). The left insula is involved in parasympathetic control, while the right insula is involved in sympathetic control (Nagai et al., 2010). The left insula is activated by pleasant emotions, such as pleasant music, while the right insula is activated by unpleasant emotions, such as various types of pain. Right insular activation has been reported in misophonia, which is an aversion or hatred to a specific sound (Schröder et al., 2019). There is an inhibitory relationship between the left and right insula that maintains sympathetic balance. Autonomic balance is said to be a physiological response to stress (Dearing et al., 2022), which indicates that the autonomic nervous system is balanced and responds to stress. Subtraction of audiovisual stimuli in ASMR also showed no significant activation of the insular cortex in the left and right insula.

We attempted to clarify the differences in mood by means of a questionnaire, but no differences were found. The 30-s ASMR video viewing may not have improved Tingling or mood due to the short duration of video viewing. Future improvements are needed, such as lengthening the video viewing time. However, the present study showed that there is a clear difference between auditory stimulation and audiovisual stimulation in terms of brain activation sites. The subjects of this study were those who had no experience with tingling, which we believe is supportive of the fact that many young people use the device for relaxation purposes regardless of tingling. However, it cannot be said that the non-experienced tinglers were accurate in their evaluation of tingling and comfort. Considering the effect on the activated areas in the brain, the mood questionnaire was also administered immediately after the MRI imaging, but it was a retrospective self-report, so further study and improvement could be needed. In the next step, we believe that the subjects also need to be compared between those who experienced tingling and those who did not. In the future, it will be necessary to evaluate stress, anxiety, and pain using physiological indices and rating scales. This could clarify the effects of ASMR on anxiety, stress, and pain by showing the relationship between physiological indices and rating scales and brain activation sites, and may indicate that ASMR is useful for mental health.

The present study has several other limitations. We believe that physiological indices and rating scales for stress, anxiety, and pain could also be investigated to clarify differences in mood improvement and physiological responses. In this study, there was no control group, such as non-ASMR. Comparison of relaxing videos may be useful to further elucidate the unique nature of ASMR. In the future, a three-way comparison between non-ASMR, sound-only ASMR, and moving image ASMR should be conducted simultaneously. In addition, it will be necessary in the future to analyze and examine whether there is a functional relationship between the characteristic activation sites at the network level. Also, the number of ASMR types was quite limited to 5. Preference for ASMR is largely a matter of personal choice, and a wider selection might have yielded different characteristics and results. We would like to consider further increasing the number of participants. The age range was also limited to young people, and the results may differ if a wider range of age groups is targeted. Further experiments targeting older adults are needed.

## 5. Conclusion

The results of this study showed that there was a clear difference between auditory and audiovisual stimulation in terms of the areas of activation in the brain, but the questionnaire did not reveal any difference in the subjects’ mood. Audiovisual stimulation showed activation of the middle frontal gyrus and the nucleus accumbens, whereas auditory stimulation showed activation of the insular cortex. The difference in brain activation sites suggests a difference in the effects of auditory and audiovisual stimulation. However, in the future, it will be necessary to investigate the relationship between the brain activation sites by comparing the effects of ASMR on those who have experienced tingling and those who have not, and by investigating physiological indices. This will clarify the effects of ASMR on depression, anxiety, stress, and pain, and may indicate that ASMR is useful for mental health.

## Data availability statement

The original contributions presented in this study are included in this article/supplementary material, further inquiries can be directed to the corresponding author.

## Ethics statement

The studies involving human participants were reviewed and approved by the Research Ethics Committee of Niigata University of Health and Welfare. The patients/participants provided their written informed consent to participate in this study.

## Author contributions

NS and NK conceived the study and designed the experiments. NS, KN, ST, and NK collected MR data and performed statistical analysis. NS and KN performed data interpretation. SK, HO, and NK helped draft the manuscript. All authors approved the final version of the submitted manuscript.
